# Anterior Mediastinal Mature Teratoma Presenting With Mediastinal Mass Syndrome in a Six‐Month‐Old Infant: Diagnostic Pitfalls and Anesthetic Challenges

**DOI:** 10.1002/ccr3.73618

**Published:** 2026-09-25

**Authors:** Ghamar Taj Khanbabaee, Ali Shafiee, Zahra Ghomi, Nastaran Sadat Mahdavi, Leili Mohajerzadeh, Yalda Nilipour, Seyed Alireza Mahdavi

**Affiliations:** ^1^ Department of Pulmonology, Mofid Children's Hospital Shahid Beheshti University of Medical Sciences Tehran Iran; ^2^ Department of Radiology, Mofid Children's Hospital, School of Medicine Shahid Beheshti University of Medical Sciences Tehran Iran; ^3^ Department of Anesthesiology, Mofid Children's Hospital, School of Medicine Shahid Beheshti University of Medical Sciences Tehran Iran; ^4^ Pediatric Surgery Research Center, Research Institute for Children's Health Shahid Beheshti University of Medical Sciences Tehran Iran; ^5^ Pediatric Pathology Research Center, Research Institute for Children's Health Shahid Beheshti University of Medical Sciences Tehran Iran; ^6^ Anesthesiology Research Center Shahid Beheshti University of Medical Sciences Tehran Iran

**Keywords:** anterior mediastinal mass, infant, mature teratoma, mediastinal mass syndrome, pediatric anesthesia

## Abstract

An anterior mediastinal mass in infants can present with life‐threatening complications due to airway and vascular compression. We report a six‐month‐old infant with a mature teratoma presenting as mediastinal mass syndrome, highlighting the diagnostic challenges and anesthetic risks involved. The patient developed significant respiratory compromise, underscoring the importance of early recognition and cautious perioperative management. Imaging played a central role in diagnosis, while perioperative planning required special consideration because of the risk of airway collapse under anesthesia. This case reinforces the need for heightened clinical suspicion and multidisciplinary coordination when managing mediastinal masses in infants.

## Key Clinical Message

Anterior mediastinal teratoma in infants may cause life‐threatening mediastinal mass syndrome. Sedation and general anesthesia can precipitate catastrophic airway and cardiovascular collapse. Early recognition and multidisciplinary perioperative planning are essential for safe management.

## 1 Introduction

Anterior mediastinal masses are uncommon in infancy, and teratomas, lymphomas, and thymic lesions account for most of the pathological entities encountered in this compartment [1, 2]. Benign thymic enlargement is also frequently seen at this age and can closely mimic mediastinal widening on plain radiography, which may lead clinicians to overlook a true underlying mass [3]. Large anterior mediastinal tumors can compress the airway, heart, and great vessels, predisposing affected infants to sudden respiratory or cardiovascular collapse, particularly during sedation, anesthesia, or changes in body position [4, 5].

The clinical picture produced by a mediastinal mass is dictated largely by its size, location, and the structures it compresses. Airway compression typically manifests as cough, wheeze, stridor, or positional dyspnea, whereas compression of the superior vena cava (SVC) or the heart itself produces facial or upper‐body swelling, plethora, and reduced cardiac output [4, 5]. Because these symptoms often overlap with common respiratory illnesses of infancy, an underlying mass may go unrecognized until compression becomes critical, as occurred in the case reported here.

Mediastinal mass syndrome represents a potentially fatal complication characterized by acute airway obstruction and hemodynamic compromise, occurring most often when spontaneous respiration and airway tone are lost during induction of general anesthesia [6, 7, 8]. Careful diagnostic evaluation and meticulous anesthetic planning are therefore essential, especially in infants with large mediastinal lesions.

Teratomas are germ cell tumors composed of tissues derived from more than one embryonic layer. Although most commonly gonadal in origin—arising in the ovaries or testes—and also encountered in the sacrococcygeal region and retroperitoneum, teratomas may occasionally arise within the mediastinum, where the anterior compartment is by far the most frequent site [1, 9]. Ovarian teratomas, in particular, are well recognized not only as space‐occupying gonadal masses but also as a trigger of paraneoplastic anti‐N‐methyl‐D‐aspartate receptor encephalitis, illustrating that the clinical relevance of this tumor type extends well beyond its site of origin [9].

We report a 6‐month‐old infant with a giant anterior mediastinal mature teratoma initially misinterpreted as a thymic shadow, who subsequently developed mediastinal mass syndrome and required emergent surgical intervention.

## 2 Case History/Examination

**FIGURE 1:**
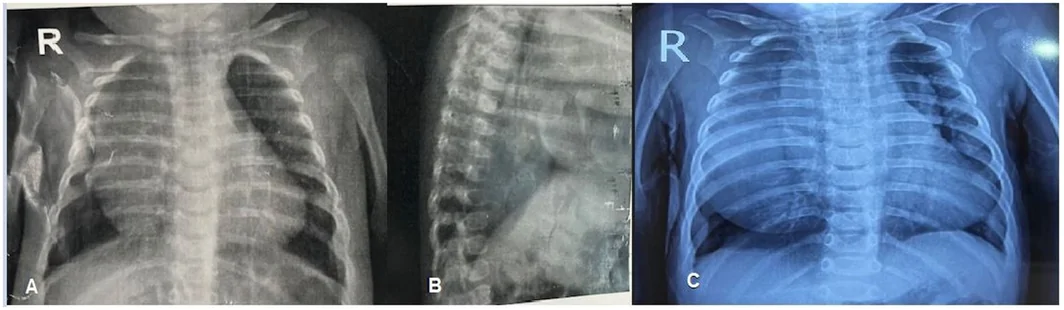
Chest radiographs at 4 and 6 months of age. (A) Anteroposterior (AP) supine view at 4 months, demonstrating mediastinal widening initially interpreted as a prominent thymic shadow. (B) Lateral view at 4 months, showing increased anterior mediastinal soft‐tissue density. (C) AP supine view at 6 months, showing marked progression of the mediastinal widening, with mass effect and decreased aeration of the right lung. On retrospective review, the contour and extent of the opacity in (A) and (B) are atypical for physiologic thymic enlargement; the interval progression seen in (C) is highly suggestive of an underlying anterior mediastinal mass rather than simple thymic prominence, illustrating a diagnostic pitfall in infancy.

A 6‐month‐old infant presented with persistent tachypnea since 2 months of age. Initial chest radiography at 4 months of age demonstrated mediastinal widening, which was interpreted as physiologic thymic enlargement (Figure 1). At 6 months of age, worsening respiratory distress prompted referral to a tertiary care center; follow‐up chest radiography demonstrated marked progression of the mediastinal widening (Figure 1). Several hours after chest computed tomography (CT) imaging, the patient developed irritability, facial puffiness, and apnea that improved with repositioning and supplemental oxygen, raising concern for mediastinal mass syndrome.

## 3 Differential Diagnosis, Investigations, and Treatment

**FIGURE 2:**
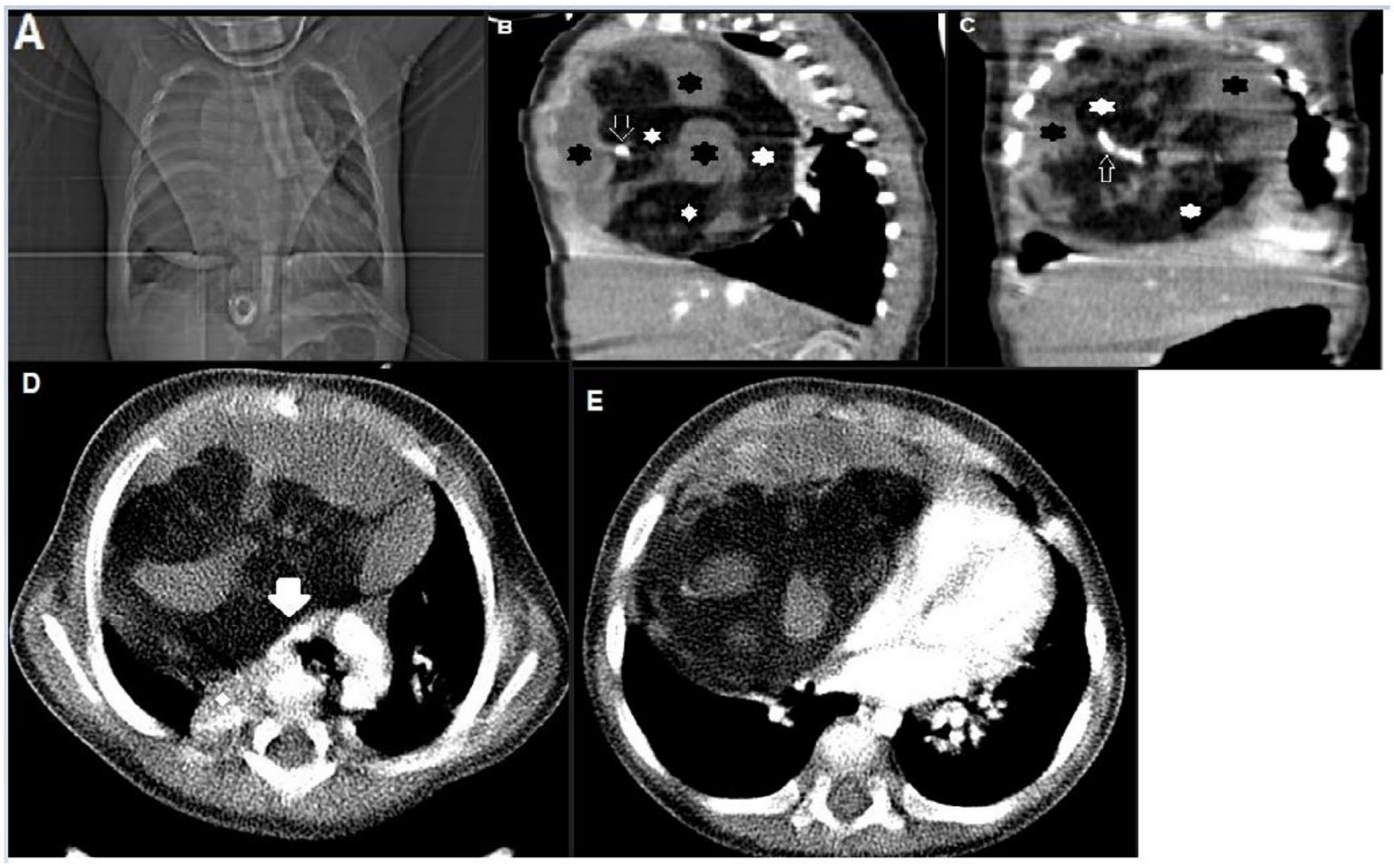
Contrast‐enhanced computed tomography (CT) of the chest. Scout image (A) shows a large mediastinal mass. Sagittal (B) and coronal (C) reconstructed images show a large anterior mediastinal mass with fat (white stars), cystic (black stars), and calcified (white empty arrows) components, compatible with teratoma. Axial images (D, E) demonstrate compression of the superior vena cava (SVC; white solid arrow in D) and the right heart (E). Collapse of the posterior segment of the right upper lobe (RUL; white solid diamond) is evident due to compression of its segmental bronchus.

**FIGURE 3:**
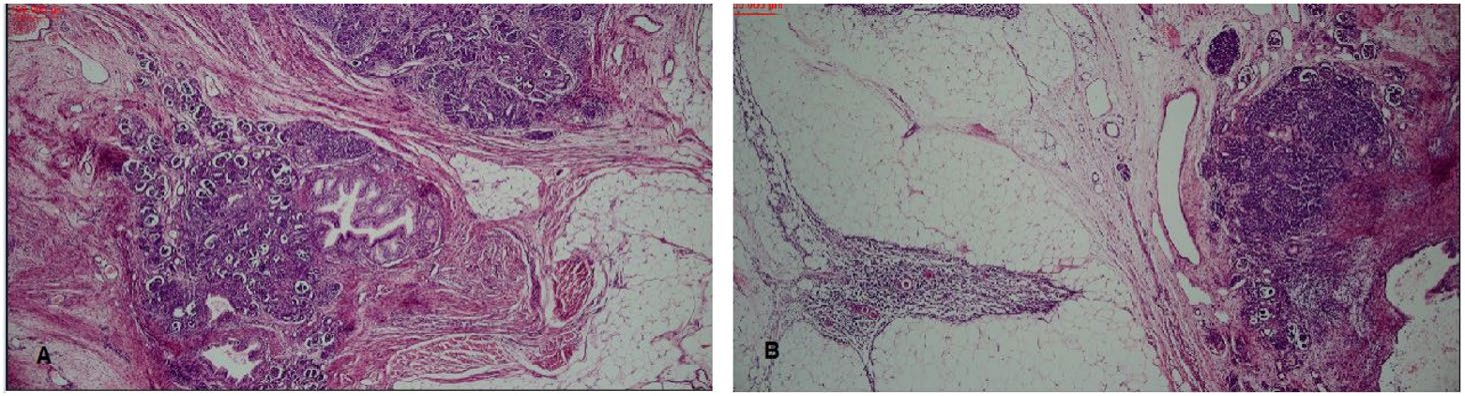
Representative histopathological features of the mediastinal tumor. (A) Tumor composed of multiple mature tissue elements, including renal tissue, mucinous glandular structures, adipose tissue, and both smooth and skeletal muscle. (B) Tumor tissue adjacent to thymic tissue, demonstrating the close anatomical association between the mass and the native thymus. Additional representative sections (hyaline cartilage with nephrogenic elements; low‐power tumor–thymus interface) are available from the corresponding author upon request.

Differential diagnosis included thymic enlargement, lymphoma, germ cell tumor, and other anterior mediastinal masses [1, 4, 5]. Contrast‐enhanced CT demonstrated a large anterior mediastinal mass containing fat and calcifications, findings highly suggestive of mature teratoma (Figure 2). The mass compressed the SVC and the right heart and caused collapse of the posterior segment of the right upper lobe (RUL) through compression of its segmental bronchus. Given the high risk of airway and cardiovascular collapse, a carefully tailored anesthetic strategy was adopted, with preservation of spontaneous respiration and avoidance of muscle relaxants before tumor resection. Urgent right thoracotomy was performed with complete excision of the tumor. Histopathological evaluation confirmed a mature mediastinal teratoma composed of mature tissues derived from all three germ layers (Figure 3).

## 4 Anesthetic and Surgical Management

Given the large mediastinal mass and the high risk of airway and cardiovascular collapse, a carefully tailored anesthetic strategy was adopted. Two units of cross‐matched packed red blood cells were prepared preoperatively.

After standard monitoring was established, anesthesia was induced by inhalation using sevoflurane, avoiding intravenous induction. Once an adequate depth of anesthesia was achieved, a left femoral central venous line was placed under ultrasound guidance, followed by a right femoral arterial line for invasive blood pressure monitoring. Ketamine (10 mg) and atropine (0.5 mg) were administered intravenously. The trachea was intubated with an uncuffed 4.0‐mm endotracheal tube while spontaneous respiration was preserved; muscle relaxants were deliberately withheld at this stage.

Following intubation, sevoflurane was discontinued and anesthesia was maintained with isoflurane. The patient was positioned in the left lateral decubitus position and handed over to the surgical team. After complete tumor resection, a neuromuscular blocking agent was administered, and the patient was transitioned to controlled mechanical ventilation.

The procedure, performed via right thoracotomy, lasted from 12:30 p.m. to 3:00 p.m. Intraoperatively, the patient received 100 mL of packed red blood cells and 400 mL of normal saline. A well‐encapsulated mass measuring approximately 20 × 5 cm was completely excised. A right‐sided chest tube was placed, and the right lung re‐expanded satisfactorily.

## 5 Pathological Findings

Gross examination revealed an oval, tan‐colored mass measuring 10 × 7.5 × 6 cm, partially covered by an opened capsule, with a cystic area containing clear fluid. Microscopically, the lesion was composed of mature elements derived from all three germ layers, including neuroglial tissue, adipose tissue, hyaline cartilage, skeletal muscle, respiratory‐type epithelium, and mucinous gastric‐type glands. Structures resembling ovarian follicles and glomeruli were also identified. Focal nephrogenic rests were present within the tumor, and adjacent thymic tissue was involved. Immunohistochemistry showed positivity for Wilms tumor 1 (WT1) protein. These findings were consistent with a mature mediastinal teratoma (Figure 3).

## 6 Discussion

Teratomas are among the most common mediastinal tumors in children, typically arising in the anterior compartment [1, 2]. Outside the mediastinum, teratomas occur most frequently in the ovaries and testes; ovarian teratomas are additionally recognized as a paraneoplastic trigger of anti‐NMDA receptor encephalitis, illustrating that the clinical relevance of this tumor type extends well beyond its site of origin [9]. Despite their benign histology, large mediastinal lesions may cause significant mass effect, leading to respiratory distress and cardiovascular compromise through direct compression of the airway, heart, and great vessels [4, 5]. In infants, the presence of a prominent, normally involuting thymus may confound early diagnosis, as occurred in this case, in which mediastinal widening was initially attributed to physiologic thymic tissue rather than a true mass [3].

Mediastinal mass syndrome represents a critical perioperative risk, particularly following sedation or general anesthesia. Loss of spontaneous respiration, supine positioning, and positive‐pressure ventilation reduce the caliber of the already compressed airway and impair venous return and may precipitate abrupt cardiorespiratory collapse [6, 7, 8]. Preservation of spontaneous breathing, avoidance of muscle relaxants prior to tumor decompression, and establishment of femoral vascular access—as adopted in the present case—are key components of a safe anesthetic strategy, consistent with previously reported approaches to infantile anterior mediastinal teratoma (Table 1) [10, 11, 12, 13].

**TABLE 1:** Summary of previously reported infants with anterior mediastinal teratoma and airway/cardiovascular compromise, compared with the present case.

Study	Age	Presenting features	Anesthetic strategy	Outcome
Present case	6 months	Progressive tachypnea; facial puffiness and apnea consistent with mediastinal mass syndrome	Inhalational induction, spontaneous ventilation preserved, femoral central/arterial access, muscle relaxant withheld until after resection	Extubated POD1; discharged POD5; asymptomatic at 3‐month follow‐up
Brenn et al. [10] (2018)	5 months	Anterior mediastinal teratoma with airway compromise	One‐lung ventilation strategy adopted to protect against carinal/bronchial compression	Successful perioperative management using an alternative airway strategy
Razafimanjato et al. [12] (2019)	5 months (girl)	Recurrent infectious pneumonia and hemoptysis	Anesthetic and surgical strategy individualized to tumor size, reviewed alongside prior literature	Favorable outcome reported
Howell et al. [11] (2022)	2 months (girl)	Cough, rhinorrhea, noisy breathing; cardiac displacement on imaging	Deliberate right‐mainstem intubation without paralysis; sternotomy and peripheral cannulation access prepared in advance	Uncomplicated course; extubated POD1; discharged POD5
Adam et al. [13] (2024)	3 months	Cyanosis and respiratory distress from a giant congenital teratoma (tumor weight twice the infant's body weight)	Staged approach: sedation for CT imaging and percutaneous drainage, followed by thoracotomy with epidural analgesia for definitive resection	Extubated in the operating room after resection

Abbreviation: POD, postoperative day.

As summarized in Table 1, previously reported infants with large anterior mediastinal teratomas share several recurring features with the present case: nonspecific respiratory symptoms preceding diagnosis, a preference for spontaneous‐ventilation induction techniques with muscle relaxants withheld until the surgical team is ready to intervene, and a generally favorable outcome when these principles are followed [10, 11, 12, 13]. This consistency across independently reported cases reinforces the practical value of a standardized, multidisciplinary approach to this rare but potentially catastrophic presentation.

This case emphasizes the need for heightened clinical suspicion in infants with persistent tachypnea and mediastinal widening on imaging, careful distinction of pathological masses from physiologic thymic tissue, and multidisciplinary planning among pediatric pulmonology, radiology, anesthesiology, and surgery to prevent catastrophic anesthetic complications.

## 7 Conclusion and Results (Outcome and Follow‐Up)

The patient was successfully extubated on postoperative day one and discharged in good condition on postoperative day five. At 3‐month follow‐up, the infant remained asymptomatic with normal respiratory status. This case highlights the importance of early recognition of mediastinal masses in infants—with careful distinction from physiologic thymic enlargement—and the critical role of multidisciplinary perioperative planning in preventing catastrophic anesthetic complications.

## Author Contributions

**Ghamar Taj Khanbabaee:** conceptualization, supervision, writing – review and editing. **Ali Shafiee:** conceptualization, data curation, writing – original draft, investigation, writing – review and editing. **Zahra Ghomi:** writing – review and editing, investigation, visualization. **Nastaran Sadat Mahdavi:** writing – review and editing, investigation. **Leili Mohajerzadeh:** supervision, writing – review and editing. **Yalda Nilipour:** writing – review and editing, investigation. **Seyed Alireza Mahdavi:** supervision, writing – review and editing, investigation.

## Funding

The authors have nothing to report.

## Ethics Statement

This case report was conducted in accordance with institutional ethical standards.

## Consent

Written informed consent was obtained from the patient's legal guardians for publication of this case and accompanying images.

## Data Availability

The data that support the findings of this study are available on request from the corresponding author. The data are not publicly available due to privacy or ethical restrictions.
